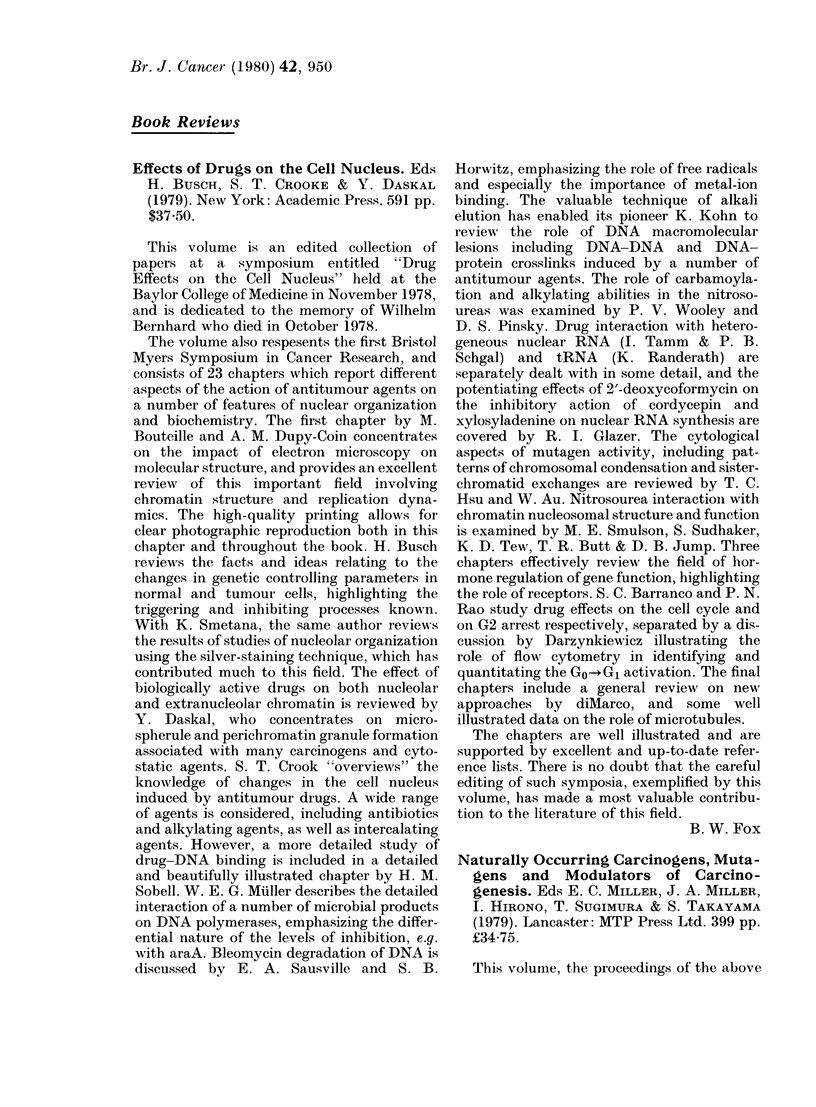# Effects of Drugs on the Cell Nucleus

**Published:** 1980-12

**Authors:** B. W. Fox


*Br. J. Cancer* (1980) **42**, 950

### Book Reviews

**Effects of Drugs on the Cell Nucleus.** Eds H. BUSCH, S. T. CROOKE & Y. DASKAL (1979). New York: Academic Press. 591 pp. $37·50.

This volume is an edited collection of papers at a symposium entitled "Drug Effects on the Cell Nucleus" held at the Baylor College of Medicine in November 1978, and is dedicated to the memory of Wilhelm Bernhard who died in October 1978.

The volume also respesents the first Bristol Myers Symposium in Cancer Research, and consists of 23 chapters which report different aspects of the action of antitumour agents on a number of features of nuclear organization and biochemistry. The first chapter by M. Bouteille and A. M. Dupy-Coin concentrates on the impact of electron microscopy on molecular structure, and provides an excellent review of this important field involving chromatin structure and replication dynamics. The high-quality printing allows for clear photographic reproduction both in this chapter and throughout the book. H. Busch reviews the facts and ideas relating to the changes in genetic controlling parameters in normal and tumour cells, highlighting the triggering and inhibiting processes known. With K. Smetana, the same author reviews the results of studies of nucleolar organization using the silver-staining technique, which has contributed much to this field. The effect of biologically active drugs on both nucleolar and extranucleolar chromatin is reviewed by Y. Daskal, who concentrates on microspherule and perichromatin granule formation associated with many carcinogens and cytostatic agents. S. T. Crook "overviews" the knowledge of changes in the cell nucleus induced by antitumour drugs. A wide range of agents is considered, including antibiotics and alkylating agents, as well as intercalating agents. However, a more detailed study of drug–DNA binding is included in a detailed and beautifully illustrated chapter by H. M. Sobell. W. E. G. Müller describes the detailed interaction of a number of microbial products on DNA polymerases, emphasizing the differential nature of the levels of inhibition, *e.g.* with araA. Bleomycin degradation of DNA is discussed by E. A. Sausville and S. B. Horwitz, emphasizing the role of free radicals and especially the importance of metal-ion binding. The valuable technique of alkali elution has enabled its pioneer K. Kohn to review the role of DNA macromolecular lesions including DNA–DNA and DNA–protein crosslinks induced by a number of antitumour agents. The role of carbamoylation and alkylating abilities in the nitrosoureas was examined by P. V. Wooley and D. S. Pinsky. Drug interaction with heterogeneous nuclear RNA (I. Tamm & P. B. Schgal) and tRNA (K. Randerath) are separately dealt with in some detail, and the potentiating effects of 2′-deoxycoformycin on the inhibitory action of cordycepin and xylosyladenine on nuclear RNA synthesis are covered by R. I. Glazer. The cytological aspects of mutagen activity, including patterns of chromosomal condensation and sister-chromatid exchanges are reviewed by T. C. Hsu and W. Au. Nitrosourea interaction with chromatin nucleosomal structure and function is examined by M. E. Smulson, S. Sudhaker, K. D. Tew, T. R. Butt & D. B. Jump. Three chapters effectively review the field of hormone regulation of gene function, highlighting the role of receptors. S. C. Barranco and P. N. Rao study drug effects on the cell cycle and on G2 arrest respectively, separated by a discussion by Darzynkiewicz illustrating the role of flow cytometry in identifying and quantitating the $G_0 \rightarrow G_1$ activation. The final chapters include a general review on new approaches by diMarco, and some well illustrated data on the role of microtubules.

The chapters are well illustrated and are supported by excellent and up-to-date reference lists. There is no doubt that the careful editing of such symposia, exemplified by this volume, has made a most valuable contribution to the literature of this field.

B. W. FOX